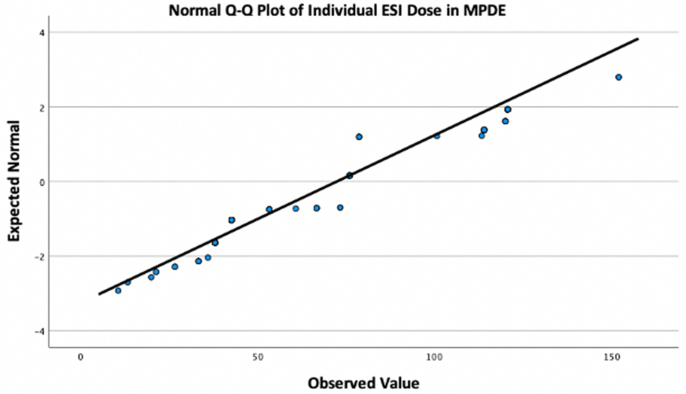# Cumulative glucocorticoid exposure in patients receiving epidural steroid injections: A single-centre retrospective evaluation on 581 procedures against existing clinical recommendations

**DOI:** 10.1016/j.inpm.2022.100094

**Published:** 2022-05-05

**Authors:** Kate Brown-Beresford, Medhat Wahba, Peter Herriot, Georgia Smithson-Tomas, Venkatesan Thiruvenkatarajan

**Affiliations:** a Pain Management Unit, Flinders Medical Centre, Adelaide, South Australia, Australia; b Department of Anaesthesia, The Queen Elizabeth Hospital, Adelaide, South Australia, Australia; c Discipline of Acute Care Medicine, University of Adelaide, South Australia, Australia

**Keywords:** Glucocorticoids, Epidural pain interventions, Non-epidural pain interventions, Postmenopausal women, Steroid dose exposure

## Abstract

**Background:**

The purpose of the study was to review the cumulative corticosteroid doses received from epidural and non-epidural-based pain interventions in a cohort of patients undergoing epidural steroid injections (ESIs) with comparison to safe dosing recommendations.

**Methods:**

Retrospective analysis was undertaken for all 349 patients who underwent a total of 581 ESIs at a single-centre, tertiary hospital in South Australia between 2017 and 2019. The primary outcome was the yearly dose analysis of cumulative steroid doses in methylprednisolone equivalents (MDPE) administered from epidural and non-epidural interventions in post-menopausal women, interpreted against maximum recommended doses.

**Results:**

The annual limit of 200 ​mg for postmenopausal women was exceeded in 4.7% of the time (11/235) from ESIs alone, with a significant rise to 15.3% (46/300) when non-ESI injections were included in cumulative dose totals(p ​< ​0.001). Of the 173 participants of post-menopausal female age, 4.1% (7/173) received cumulative corticosteroid doses above the 3-year 400 ​mg MPDE limit from ESIs alone, with a statistically significant increase to 13.9% (24/173) when non-epidural steroid injections were again included in cumulative dose totals (p ​< ​0.001). The mean ​± ​standard deviation administered MPDE per epidural steroid injection across the whole study cohort was 72 ​± ​22 ​mg, nearly double the recommended dose of 40 ​mg.

**Conclusions:**

Our study underpins the need for vigilance when considering steroid-based pain interventions, wherein both the individual and cumulative steroid exposure should be considered.

## Highlights

• Steroid exposure from epidural- and non-epidural interventions.
• Steroid exposure in post-menopausal women.
• Risk of excess steroid exposure is a possibility from epidural and non-epidural interventions.
• Physicians should be vigilant about excess steroid exposure.

## 1 Introduction

Epidural corticosteroid injections (ESIs) are supported by current evidence for the short-term management of spinal radicular pain [1]. Despite being a widely performed procedure globally, epidural steroid administration has not been approved by either the US Food and Drug Administration (FDA) or European Medicines Agency, and hence is classified as “off-label” use [2]. The utilisation rate for these procedures is continually increasing, with an estimated 9 million ESI interventions performed annually in the US [3,4]. Australian data demonstrated an increase from 31,500 to 35,000 spinal steroid injections in a single year from 2011 to 2012, with current numbers expected to be higher [5]. Contemporary literature promotes an increased awareness of “the cumulative steroid exposure” from neuraxial as well as non-neuraxial interventions to reduce systemic side effects [6]. The literature demonstrates that an ESI of a single methylprednisolone equivalent dose (MPDE) of 80 ​mg, or cumulative doses of 200 ​mg in a single year, or 400 ​mg over 3 years, has negative consequences for bone mineral density, especially in post-menopausal women [[6], [7], [8], [9], [10]]. As such, governing bodies have endorsed the recommendation that, over 3 years, MPDE doses above 400 ​mg in post-menopausal women and 3 ​g in healthy men may impact bone mineral density or increase the risk of osteoporotic fractures [6]. Latest systematic reviews also reinforce this recommendation [9]. Excessive steroid exposure also increases the risk of hypothalamic-pituitary-adrenal (HPA) suppression and hypertension [[6], [7], [8], [9], [10]].

Recent Australian data demonstrates that oral corticosteroids are dispensed in potentially toxic cumulative doses in up to one-quarter of asthmatic patients [11]. Australian data on procedurally administered corticosteroid doses is lacking, however. Latest literature reiterates that total corticosteroid dose calculations should encompass all local steroid injections administered to the patient, due to the significant systemic side effects associated with both epidural and intraarticular injection routes [12]. It is likely that ESIs are often repeated along with other non-epidural steroid injections, and due to lack of attention, or for lack of records, physicians may not be aware if a particular patient has reached maximum safe doses. Currently, data is limited as to what extent patients are exposed to potentially harmful cumulative doses of steroids from commonly performed interventions as ESIs. We therefore conducted a single-centre retrospective study over a 3-year period in our practice to identify whether patients undergoing ESIs exceeded recommended safe cumulative doses. We hypothesised that, given the relatively recent provision of upper limit steroid dosing recommendations, there may be patients at risk of unsafe cumulative doses within our practice.

## 2 Materials and methods

All patients who underwent an ESI for spinal and radicular pain at Flinders Medical Centre Pain Management Unit in Adelaide, South Australia, between 1 January 2017 and 31 December 2019, were identified by searching the appropriate Medicare billing codes via the hospital's Operating Room Information System (ORMIS) software. Variables recorded included Unit Record (UR) number, gender, age, and date of procedure. Each individual case was reviewed and relevant data relating to the specific details of the ESI was collected through the hospital's OACIS (Open Architecture Clinical Information System) electronic information system. Additional non-ESI steroid-based pain interventions were then identified for each patient using OACIS by reviewing all other pain unit's activity for this patient cohort during this period. Any additional non-ESI steroid injection was then included in steroid dose totals (inclusive of intra-articular injections [facet joints, sacroiliac, sacrococcygeal, shoulder], nerve blocks [lumbar nerve roots, greater occipital nerve, suprascapular nerve, axillary nerve, genicular nerve, ganglion of impar, gluteal nerve, obturator nerve, infraorbital nerve, supraorbital nerve], bursal injections and additional spinal procedures in which steroids were incorporated [medial branch blocks, radiofrequency ablations/rhizolysis]). Our search methodology on OACIS and ORMIS did not allow for analysis of intramuscular and/or systemic steroid exposure, thus these were excluded. Similarly, all corticosteroid injections provided by the state's public hospital radiology services, as available via OACIS, were also reviewed and included. If a steroid-based injection was identified then details including date, type of procedure, steroid type and amount used were recorded. In several cases, the radiology provider failed to specify the steroid dose in milligrams and instead recorded millilitres. In such cases, it was assumed that betamethasone 1 ​mL was equivalent to 5.7 ​mg and dexamethasone 1 ​mL was equivalent to 4 ​mg, as per standard vial concentrations available in South Australia.

The primary outcome was the yearly dose analysis of cumulative steroid doses (MPDE) administered from epidural and non-epidural interventions in post-menopausal women, interpreted against maximum recommended doses. Cumulative dose included the total amounts of steroid administered from both ESI and additional non-ESI steroid-based injections. Recommended maximum doses are only clearly provided in the literature for the post-menopausal female cohort and are <200 ​mg in a single year or <400 ​mg over 3-years [[6], [7], [8], [9]]. Secondary outcomes were the cumulative steroid doses (MPDE) administered from epidural and non-epidural interventions in postmenopausal women across the 3-year interval, interpreted against maximum recommended doses and analysis of the cumulative steroid doses in the entire study cohort further categorised by gender (male vs female), as well as in the specific pre-menopausal female cohort.

To categorise the female patients in the study cohort into ‘pre-menopausal’ and ‘post-menopausal’ groups for the primary outcome analysis, all females aged 52 and above at the time of procedure were defined as ‘post-menopausal’, based on the Royal Australian and New Zealand College of Obstetricians and Gynaecologists (RANZCOG) Australian average age of menopause [13]. In order to standardise the administered steroid amounts across the cohort, all corticosteroid medication doses were converted to MPDE as per standard dose conversion tables, displayed in Supplemental Table 1 [14,15]. Data was collected and analysed using Microsoft Excel (Version 16 for Mac) and IBM SPSS Statistics software (Version 28 for Mac). As a convenience sample was utilised, no sample size calculation was attempted. Using SPSS, the data on administered individual ESI doses appeared normally distributed with skewness ​= ​0.287 and kurtosis ​= ​0.699, with the Q-Q plot represented in Supplemental Fig. 1 [16]. A Wilcoxon signed-rank test (2-tailed, matched pairs) was used to determine if there was a significant difference in the 3-year cumulative corticosteroid doses when non-ESI administered steroids were added to ESI dose totals. This test was used as data was paired for each patient and the distributions were determined to be non-parametric using Kolmogorov-Smirnov and Shapiro-Wilk tests of normality. A *p*-value <0.05 was considered significant. The data are presented as mean ​± ​standard deviation (SD) or median (IQR) as appropriate.

This project was assessed by the Southern Adelaide Local Health Network (SALHN) Office for Research (02.12.2020) and it was determined that ethics review and approval was not required under the National Health & Medical Research Council (NHMRC) ethical considerations in quality assurance and evaluation activities guidelines [17].

### 2.1 Results

The study cohort consisted of 349 patients (females, n ​= ​224 and males, n ​= ​125) with a mean ​± ​SD age of 64 ​± ​15 years, with further cohort demographics depicted in Table 1. The cohort underwent a total of 590 ESI and 441 additional non-ESI steroid-based procedures during the study period. A total of nine ESI procedures had inadequate documentation about administered doses and were then excluded, leaving 581 ESI procedures for further analysis. Of the 349 patients, 118 (33.8%) had more than one ESI procedure performed during the 3-year period. Within the total ESI cohort, there were 173 females of post-menopausal age.

**Table 1:** Demographics of study cohort.

Variables	Females	Males	Total
Number of patients, N (%)	224 (64.2)	125 (35.8)	349
Age (Years; M ​± ​SD)	63.9 ​± ​14.9	65.4 ​± ​15.8	64.4 ​± ​15.2
ESI procedures performed over 3 years	388	202	590
Average ESI procedures per patient over 3 years (M ​± ​SD)	1.72 ​± ​1.29	1.62 ​± ​1.40	1.69 ​± ​1.33
Non-ESI procedures performed over 3 years, N (%)	320 (72.5)	121 (27.5)	441
Average non-ESI procedures over 3yrs (mean ​± ​SD)	1.42 ​± ​1.74	0.97 ​± ​1.34	1.26 ​± ​1.63

Abbreviations: ESI, epidural steroid injections; M, mean; SD, standard deviation; N, number.

#### 2.1.1 Primary outcome

**Fig. 1:**
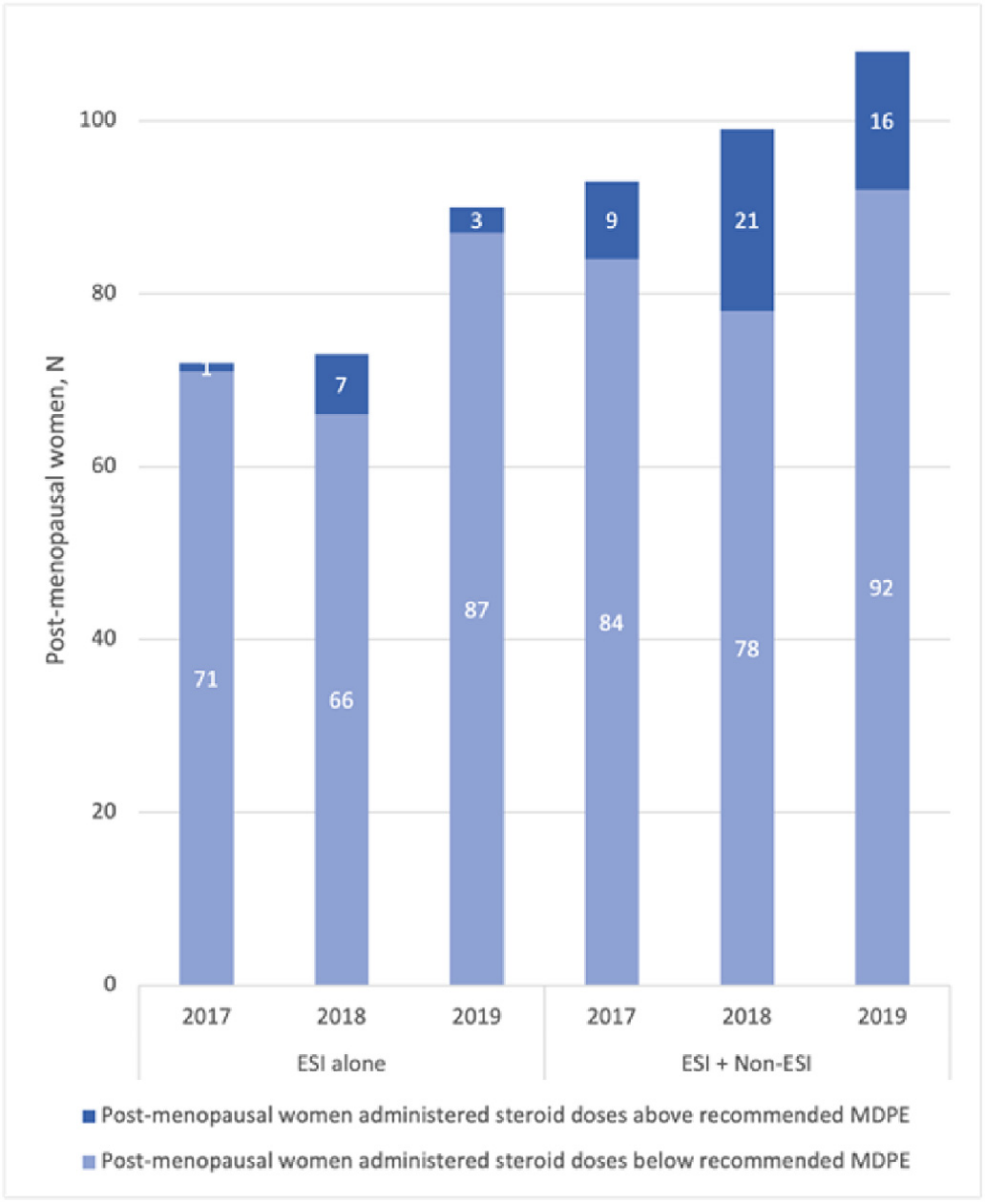
**Single-Year Analysis from 2017**–**2019 of Cumulative Steroid Doses From ESI and Non-ESI procedures in Post-Menopausal Women.** The women were represented as being either above the maximum recommended dose of <200 ​mg over a 1-year period (darker shading) or below the dose (lighter shading) Abbreviations: ESI, epidural steroid injections; MDPE, methylprednisolone dose equivalent.

Within the post-menopausal female cohort, yearly steroid exposure exceeding 200 ​mg MPDE from ESIs alone was noted in 11 out of 235 instances (4.7%), which increased significantly to 46 out of 300 (15.3%) instances when non ESI interventions were included (z ​= ​−11.064, p ​< ​0.001) (Fig. 1). The highest 3-year cumulative steroid dose received in this post-menopausal cohort was 544 ​mg MPDE via ESIs alone, and 798 ​mg MPDE from combined ESI and non-ESI procedures. Further data pertaining to this cohort is depicted in Table 2, Table 3.

**Table 2:** Descriptive data on the post-menopausal female cohort only, grouped by steroid dose received compared to the recommended maximum of 200 ​mg MPDE in a single year or 400 ​mg MPDE within a 3-year period.

SINGLE YEAR ANALYSIS: POST-MENOPAUSAL FEMALE COHORT		
	<200 ​mg MPDE	>200 ​mg MPDE
	Number of cases, N (%)	Number of cases, N (%)
**Annual ESI procedures only**		
*2017*	71 (98.61)	1 (1.39)
*2018*	66 (90.41)	7 (9.59)
*2019*	87 (96.67)	3 (3.33)
**Annual total combined steroid (ESI ​+ ​Non-ESI procedures)**		
*2017*	84 (90.32)	9 (9.68)
*2018*	78 (78.79)	21 (21.21)
*2019*	92 (85.19)	16 (14.81)
**3 YEAR ANALYSIS: POST MENOPAUSAL FEMALE COHORT**		
	**< 400 ​mg MPDE**	**> 400 ​mg MPDE**
	Number of cases, N (%)	Number of cases, N (%)
**3-year Cumulative Steroid Dose**		
*ESI only procedures*	166 (95.95)	7 (4.05)
*Non- ESI only procedures*	164 (94.80)	9 (5.20)
*Combined ESI ​+ ​Non-ESI procedures*	149 (86.13)	24 (13.87)

Abbreviations: ESI, epidural steroid injections.

**Table 3:** Descriptive statistics for administered steroid doses in MPDE (mg) to the different subgroups within the ESI patient cohort.

	Pre-menopausal Female	Post-menopausal Female	Combined Female	Male	Total
*Steroid doses per individual ESI procedure, mg, M (SD)*	82 (28)	68 (20)	71 (22)	76 (22)	72 (22)
*ESI only cumulative 3-year steroid doses, mg, MED (IQR)*	76 (43)	76 (53)	76 (45)	76 (39)	76 (44)
*ESI ​+ ​non-ESI procedure total cumulative 3-year steroid doses, mg, MED (IQR)*	119 (228)	172 (185)	161 (192)	119 9152)	152 (190)

Abbreviations: ESI, epidural steroid injections; IQR, Interquartile Range; M, Mean; MED, Median; SD, standard deviation.

#### 2.1.2 Secondary outcomes

**Fig. 2:**
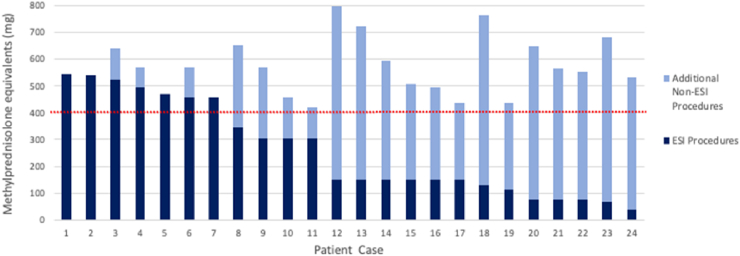
**Individual Cumulative 3-Year Steroid Doses for Cases Exceeding 400 ​mg MPDE in Post-Menopausal Women.** The cumulative 3-year steroid dose in the 24 individual post-menopausal female cases in whom the recommended 400 ​mg MPDE 3-yearly dose was exceeded. Cumulative amounts for each case are broken down into the steroid delivery via ESI procedures (dark blue) or by additional non-ESI-based procedures (light blue), with the red-dotted line depicting the 400 ​mg MPDE threshold. Abbreviations: ESI, epidural steroid injectionss

Of the 173 females of post-menopausal age, 13.9% (24/173) received cumulative corticosteroid doses above 400 ​mg MPDE over 3-years from combined ESI and non-ESI interventions. The dose characteristics of the individual cases are depicted in Fig. 2. In the same period, the proportion that received excess doses from ESI alone was 4.1% (7/173); this difference was significant [13.9% (24/173) vs 4.1% (7/173), Z −9.029, p ​< ​0.001].

**Fig. 3:**
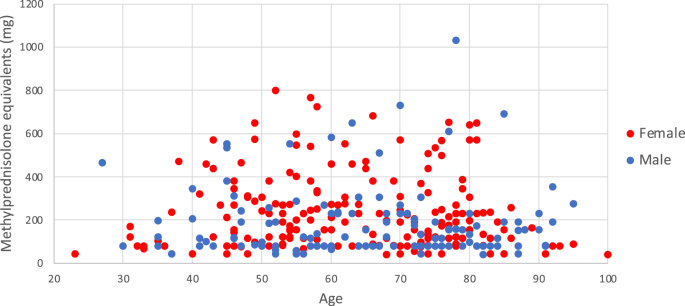
**Cumulative 3-year Steroid Amounts by Age.** Scatter plot depicting total cumulative 3-year steroid dose administered in MPDE for all patients by age (inclusive of ESI ​+ ​additional non-ESI steroid-based procedures).

Table 3 demonstrates descriptive statistics for the cumulative steroid doses received by the total study cohort, with graphical depiction of all patients displayed in Fig. 3. The mean (±SD) administered dose of steroid per ESI for the total combined genders was 72 ​± ​22 ​mg MPDE. The median [range (IQR)] 3-year cumulative dose for the whole cohort was 76 (44) mg for ESIs only, which increased to 152 (190) mg when additional non-ESI doses were included in 3-year totals; this difference was significant (z ​= ​−12.117, p ​< ​0.001).

Regarding the male-only cohort, the maximum 3-year cumulative dose administered was 646 ​mg for ESI-only administered steroids, which rose to 1029 ​mg when non-ESI steroids were added to cumulative dose totals. The maximum annual dose of cumulative steroids (ESI ​+ ​non-ESI) received by any male patient was 468 ​mg in 2017, 380 ​mg in 2018 and 418 ​mg in 2019. The total male cohort therefore remained well below of the 3 ​g over 3-years suggested upper limit for healthy males.

Regarding the female-only cohort, there were 51 females of pre-menopausal age (compared to 173 of post-menopausal age). Pre-menopausal females received a higher MPDE mean (±SD) dose of 82 ​± ​28 ​mg (per ESI), compared to post-menopausal females who received 68 ​mg ​± ​20 ​mg. However, pre-menopausal females received a lower median (IQR) 3-year cumulative total steroid dose of 119 (228) mg vs 172 (185) mg in post-menopausal females. Pre-menopausal females underwent a mean of 2 ​± ​1.2 ESI procedures and only 1 ​± ​1.4 non-ESI procedures, per patient over 3-year period, compared to 2 ​± ​1.3 ESI procedures and 2 ​± ​1.8 non-ESI procedures in the post-menopausal group. Of note, 3.9% (2/51) of pre-menopausal females received steroid doses of over 400 ​mg over 3-years from ESIs alone, which rose to 13.7% (7/51) when additional non-ESI steroids were included in cumulative dose totals.

### 2.2 Discussion

Individual dose analysis of ESI interventions showed encouraging results, as only 4% (7/173) received excess doses. However, this risk increased three times (13.8%) when non-ESI steroid doses were also included in cumulative dose totals. Similarly, 11 occurrences were identified over the 3-year period where the annual proposed safe limit of 200 ​mg MPDE was exceeded by ESIs alone. Importantly, this rose to 46 occurrences once additional non-ESI steroid injections were included in annual cumulative dose calculation. Our results revealed that roughly one in seven patients [13.9% (24/173)] in the post-menopausal age group received excessive 3-year cumulative steroid doses from combined epidural and non-epidural steroid injections.

Physicians should be vigilant about the possibility of cumulative steroid excess, with the potential for osteoporosis, HPA suppression, or hypertension [[6], [7], [8], [9], [10],^18^]. Clinicians may not have complete access to the records of all treatments received by their patients. Asking patients is a simple step that is not time-consuming, and which should be added to the standard protocol for the use of ESIs and other steroid administrations. A small change is practice is therefore warranted. However, patients may not be intimately aware of prior dosages, highlighting the utility of a “steroid pass” that collates a patient's steroid exposure, whereby electronic medical records could be leveraged to track these encounters. The “steroid pass” could also be available on a mobile application to promote patient autonomy and health literacy. Improved consent process to patients, adhering to standard minimally effective dosing with set frequencies for repeating epidural steroids, notifying general practitioners, linking radiological information of all steroid-based interventions with electronic records may mitigate the risk.

Recommendations for safe exposure limits are lacking in many patient subgroups outside the post-menopausal women and healthy men e.g., pre-menopausal women, hypo-androgenic or osteoporotic males, patients on systemic steroids, etc. Therefore, the rationale of our subgroup analysing was not to quantify the absolute cumulative steroid exposure but rather to observe the trends in steroids dosing between gender and age groups, and to assess the magnitude of change in cumulative steroid exposure when combining non-ESI procedures to ESIs.

Analysing the pre-menopausal cohort demonstrated very similar trends in those exceeding 400 ​mg in 3 years: 3.9% (2/51) with ESIs alone, up to 13.7% (7/51) with inclusion of additional non-ESI steroids. There is no evidence to support the 400 ​mg dose cut-off in pre-menopausal females; this was performed solely to further investigate dosing trends between the younger and older age cohorts. Post-menopausal females received a lower mean dose per ESI procedure compared to pre-menopausal females. Post-menopausal women, however, received higher 3-year cumulative median steroid doses and were exposed to a greater number of non-ESI steroid injections than pre-menopausal women, likely representing the increasing number of painful sites generally experienced with increasing age. Pre- and post-menopausal females received roughly the same number of annual ESI injections; however, the mean dose per ESI was lower in the post-menopausal female cohort as may be anticipated for this higher age group. The combined female cohort received a lower mean steroid dose per ESI procedure compared to the male cohort. Reassuringly, the limit of 3 ​g over 3-years in males was far from being exceeded in any patient within this cohort; nonetheless, we acknowledge that this exposure limit pertains to “healthy” men which was not examined in this review. Lastly, the mean corticosteroid dose per ESI was 72 ​mg MPDE, which is above the ‘lowest effective dose’ of 40 ​mg suggested by the World Institute of Pain Benelux Work Group [2].

The clinical significance of exceeding these upper limits of steroids predominantly revolves around diminishing bone mineral density and the associated increased risk of fragility fractures in post-menopausal women, with high associated rates of morbidity and mortality [6,7,[18], [19], [20]]. Literature demonstrates that our single mean ESI dose of 72 ​mg MPDE increases osteopenia risk by nearly 1.8% per injection [9,10]. Additionally, HPA axis suppression is another widely recognised side effect of epidural steroid administration [18]. Recent evidence supports that a dose of 20 ​mg MPDE via this route suppresses the HPA axis for 8.0 days, whilst a dose of 40 ​mg results in 19.7 days of suppression [21,22]. Habib et al. demonstrated that after an 80 ​mg MPDE dose, 17% of patients had ongoing HPA axis suppression after one month, whilst Abdul et al. similarly demonstrated eventual normalisation of the axis after 4 weeks [23,24]. The duration of HPA suppression did not appear to be dose-dependent comparing 80 ​mg and 40 ​mg MPDE, there was no significant difference between the two groups by week three [6,23]. Contrastingly, another study demonstrated that an ESI of dexamethasone 8 ​mg dose (42.7 ​mg MPDE) had no ongoing significant impact on serum cortisol and adrenocorticotropic hormone at day 7 and 21 post-injection [25]. Topically, the Spine Intervention Society recently released a statement that the new COVID-19 mRNA vaccine should not be administered within two weeks of an ESI due to this known significant HPA axis suppression [26].

There are very limited guidelines on steroid dosing limits for other non-ESI steroid-based pain management interventions. Similarly, no published recommendations exist for the combined steroid dosing limits of ESI and non-ESI injections. We found that this combination provided a statistically significant increase in cumulative steroid doses; however, it was beyond the scope of our study to determine its clinical significance. Therefore, until further evidence is available, it appears logical to combine the ESIs and non-ESIs to obtain a more accurate estimate of the cumulative systemic steroid exposure in this chronic pain patient cohort, supported by Stout et al.’s recent literature review [12].

Strengths of our research included addressing the current lack of published literature on real-life cumulative corticosteroid exposure from pain-relieving procedures in clinical pain practices. Strict methodology was employed to obtain high-quality data. Similarly, the reasonable number of cases allowed for a meaningful interpretation and a wide applicability to patients receiving spinal and other pain-relieving procedures. Study limitations included the inability to incorporate additional steroid-based procedures performed outside of our data capture range (i.e., private radiology and interstate providers), thus cumulative steroid doses may be underestimated in our cohort. This highlights the importance of establishing a centralised system for record keeping. We were also unable to assess for additional steroids administered by either the oral, inhaled, or intramuscular route. Although we defined critical steroid exposure on the recommended doses within a stipulated timeframe, it was beyond our analysis to attempt to establish a causal relationship to the extent of danger imposed by these doses. The scope of the study was restricted to dose analysis only and hence, the potential side effects linked to excess steroid exposure could not be ascertained. The cut-off age of 52 and above was utilised to assume post-menopausal status of the female cohort, which may be different to the true status, thus a possibility of a misclassification bias is a further limitation. A sensitivity analysis based on the age categories would have perhaps improved the presentation of the results. Further, the factors responsible for overexposure and the complications if any in this cohort was not analysed. Finally, as this was a single-centre study only, clinical practice may vary between centres and between proceduralists, both nationally and internationally; however, we believe our practice overall mean of 1.69 ​± ​1.33 ESI procedures per patient over a 3-year period is likely reflective of a normal, and possibly even a conservative, practice.

Due to the lack of a centralized procedural database, there is the possibility that our clinicians may have not been fully aware of steroid doses delivered previously, resulting in unintentional overdosing. Careful history taking by proceduralists and clear documentation of all steroid-based interventions by primary care providers in patient health summaries are warranted. Physicians should always consider a safety margin when calculating such cumulative doses.

Suggestions for future clinical practice include the development of a centralized database/spreadsheet for specialist interventional pain units to allow cumulative steroid amounts to be easily reviewed prior to proceeding with additional interventions (both ESI and non-ESI), marketed as a “steroid pass”. This would allow easier identification of patients at higher risk for steroid-related complications with implementation of appropriate clinical action. For example, post-menopausal women that derive significant analgesic or functional benefits from ongoing steroid injections could therefore be flagged to their primary care provider to also consider bone mineral density testing, commencement of antiresorptive therapy, or referral to fragility clinics as indicated. Similarly, some evidence suggests that regular monitoring of adrenal function in patients who have received long-term ESIs may be prudent [27]. While the risk of excess steroid exposure is acknowledged, a risk-benefit trade-off tailored to the individual case should be considered. Provision of written information to patients, including dose and date, is also recommended. Evidence supports that additional “stress dose” of steroids may be required in these patients if they were to need surgery; thus, perioperative teams should be aware [12,28]. Informing primary care providers of the above may also prevent multiple referrals to different providers, minimising inadvertent administration of excessive cumulative steroids doses in these at-risk patients.

## 3 Conclusion

Over a 3-year period, yearly-dose analysis of steroid exposure revealed recommended doses from epidural injections alone were exceeded in nearly one in twenty of our post-menopausal, which increased significantly to more than one in six when non-epidural interventions were included. Interventional pain physicians should enquire about additional steroid exposures from other possible routes and additional centres/providers and calculate the cumulative exposure before contemplating steroid injections. These principles should be reinforced and incorporated during training programs across domains including pain medicine and interventional radiology. Transparent and easily accessible documentation of cumulative steroid doses, along with enhanced primary care provider liaison, particularly regarding routine bone-mineral density and HPA axis screening as well as antiresorptive therapy in those at-risk may improve service delivery.

## Declaration of funding

This research did not receive any specific grant from funding agencies in the public, commercial, or not-for-profit sectors.

## Declaration of competing interest

The authors declare that they have no known competing financial interests or personal relationships that could have appeared to influence the work reported in this paper.

## Figures and Tables

**figs1:**