# Supplementary material for: Cumulative glucocorticoid exposure in patients receiving epidural steroid injections: A single-centre retrospective evaluation on 581 procedures against existing clinical recommendations
Source: Interv Pain Med. 2022 May 5;1(2):100094. doi: 10.1016/j.inpm.2022.100094 (PMC11372952; doi:10.1016/j.inpm.2022.100094)
Supplement: Multimedia component 1 [file mmc1.docx]

**Supplemental Table 1.** Glucocorticoid dose equivalents in milligrams for common epidurally administered steroid agents, adapted from Medscape and the Spine Intervention Society (Farinde, 2021; Mattie and Smith 2019).

| **Agent** | **Equivalent glucocorticoid doses (mg)** |
| --- | --- |
| Betamethasone | 12 |
| Dexamethasone | 15 |
| Methylprednisolone | 80 |
| Triamcinolone | 80 |
| Prednisolone | 100 |
